# Association of Platelet Membrane Glycoprotein HPA-2a/b, GP VI T13254C, and GP Ib*α* VNTR Polymorphisms with Risk of Coronary Artery Disease: A Meta-Analysis

**DOI:** 10.1155/2017/1538750

**Published:** 2017-05-18

**Authors:** Wei Ni, Jidong He, Haoyu Wang, Tao Liu

**Affiliations:** ^1^Department of Cardiology, Nanchong Central Hospital, The Second Clinical Medical College of North Sichuan Medical College, Nanchong 637000, China; ^2^Department of Cardiology, Bazhong Traditional Chinese Medical Hospital, Bazhong 636600, China

## Abstract

**Background and Aims:**

Recently, controversial results have been reported regarding the association of the polymorphisms of platelet membrane glycoproteins (HPA-2a/b, GP VI T13254C, and GP Ib*α* VNTR) with coronary artery disease (CAD). We performed this meta-analysis to further assess the polymorphisms of platelet membrane glycoproteins with a risk of CAD.

**Methods:**

A systematic electronic literature search was conducted in Embase, Cochrane Library, PubMed, and the Chinese Biomedical Literature Database (CBM). Analyses were performed using the Cochrane software package Review Manager 5.2 and Stata 12.0 software package.

**Results:**

Twenty-nine full-text articles were included in the meta-analysis. Based on random-effects meta-analysis, a significant association between the HPA-2a/b polymorphism and CAD was identified (allele model: odds ratio = 1.43, 95% confidence interval = 1.07–1.91; dominant genetic model: odds ratio = 1.57, 95% confidence interval = 1.08–2.28). Our study showed no association between the GP VI T13254C polymorphism and CAD in either a random-effects model or a fixed-effects model. Furthermore, there was no evidence to suggest that the GP Ib*α* VNTR polymorphism was associated with CAD in any of the genetic analysis models.

**Conclusions:**

The HPA-2a/b polymorphism correlated significantly with a risk of CAD, and the HPA-2b allele and the HPA-2ab + HPA-2bb genotype may increase the risk of CAD. There was no evidence to suggest that polymorphisms of GP VI T13254C and GP Ib*α* VNTR were associated with CAD.

## 1. Introduction

Coronary artery disease (CAD), one of the leading causes of mortality, is a complex disease that is multifactorial and clearly influenced by environmental factors and genetic predisposition. Platelets and thrombosis play a pivotal role in the pathogenesis of atherogenesis in CAD [1]. Platelet membrane glycoproteins Ib-IX-V, Ia-IIa, and IIb-IIIa mediated crucial reactions in the process of platelet adhesion, aggregation, and thrombosis [2]. The polymorphisms of platelet membrane glycoproteins affect the function of platelets in the process of thrombosis, and this can ultimately lead to different risks for CAD [3].

Many studies have focused on exploring the correlation between polymorphisms of platelet membrane glycoproteins and CAD; but unfortunately there has not been some kind of unified understanding [4–9]. So far, no published studies have specifically explored the association of platelet membrane glycoprotein (HPA-2a/b, GP VI T13254C, and GP Ib*α* VNTR) polymorphisms with the risk of CAD. The present meta-analysis attempts to use all relevant evidence to answer the question of whether the polymorphisms of platelet membrane glycoproteins HPA-2a/b, GP VI T13254C, and GP Ib*α* VNTR are associated with a risk of CAD.

## 2. Materials and Methods

### 2.1. Literature Search

We initially identified published studies that investigated the association of polymorphisms of platelet membrane glycoproteins (HPA-2a/b, GP VI T13254C, and GP Ib*α* VNTR) with a risk of CHD by searching the Embase, Cochrane Library, PubMed, and CBM databases from their inception through December 31, 2016. The following search terms were used: “platelet glycoprotein” or “GP I b-IX Complex” or “GP I b*α*” or “platelet membrane glycoprotein VI” or “HPA-2” or “GP I b*α*VNTR” or “GP6” AND “polymorphism or gene or variant or mutation” AND “coronary heart disease” or “CAD” or “myocardial infarction” or “angina” or “ischemic heart disease” or “ischemic cardiomyopathy” or “coronary artery stenosis”. We also reviewed the reference lists of the retrieved articles and recent reviews.

### 2.2. Study Selection

We included any study that met all of the following criteria: (1) the study was a cross-sectional, case control, or cohort design; (2) clear diagnostic criteria for CAD were established; (3) the association between of HPA-2a/b, GP VI T13254C, and GP Ib*α* VNTR with risk of CHD was investigated; and (4) the odds ratios (ORs)/risk ratio (RR, for cohort studies) and the corresponding 95% confidence intervals (CIs), or the distributions of genotype and allele frequency were reported. Two authors independently evaluated the eligibility of all studies retrieved from the databases. Disagreements were resolved by discussion or in consultation with a third author.

### 2.3. Data Extraction

Two investigators independently extracted data on patient and study characteristics and the quality of each study using a standardized data extraction form. Any discrepancies were resolved by consensus. The following information was extracted from each study: first author, year of publication, location, race, diagnostic criteria, gene, mean age, disease definition, gender, source of controls, number of patients, effect size (OR, HR), and adjusted variables.

### 2.4. Data Analysis

Statistical analyses were performed using the Cochrane software package Review Manager 5.2 and the Stata 12.0 software package. Descriptive statistics were calculated for all demographic and clinical characteristics of the study subjects. Quantitative clinical data were compared between patients with CAD and control subjects using an unpaired Student's *t*-test. Qualitative data were compared by the chi-square test. Allele frequencies were estimated by the gene counting method, and the chi-square test was used to identify significant departures from the Hardy-Weinberg equilibrium. The strength of the association between the HPA-2a/b, GP VI T13254C, and GP Ib*α* VNTR polymorphisms with risk of CAD was measured by odds ratios (ORs) and 95% confidence intervals (CIs).

The OR values of the two-allele polymorphism were calculated from the following two models, respectively: (1) allele model (mutant alleles versus wild-type alleles); (2) dominant model (heterozygous + homozygous mutant versus homozygous wild-type). The OR values of the variable number of tandem repeat (VNTR) polymorphism were calculated from the following three models, respectively: (1) allele model (single allele versus other alleles); (2) dominant model (contained a specific allele genotype versus other genotypes); (3) codominant model (single genotype versus other genotypes).

Heterogeneity was quantified using the *I*^2^ statistic. Where the *I*^2^ value was 50% or lower, indicating no evidence of heterogeneity, the fixed-effects model was suggested; otherwise, we used the random-effects model. In the presence of heterogeneity, we performed subgroup and sensitivity analyses to explore possible explanations for the heterogeneity and examine the influence of various exclusion criteria on the overall risk estimate. The funnel plot and Egger's test were conducted to identify a potential publication bias.

## 3. Results

### 3.1. Study Selection

**Figure 1:**
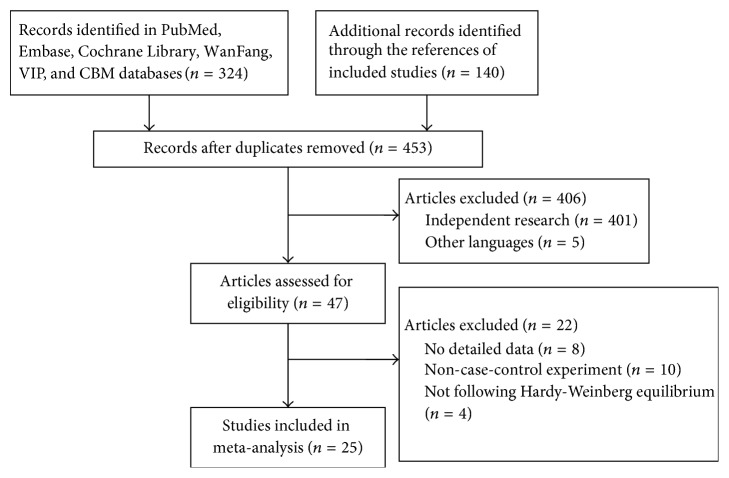
Flow chart from identification of eligible studies to final inclusion.

A flowchart of the study selection process is shown in Figure 1. Twenty-five published studies (containing 29 research investigations) were finally included in the meta-analysis [4–28]. Among these, one study contained two independent researches [13], and three studies explored HPA-2a/b and GP Ib*α* VNTR, simultaneously [5, 11, 16]. In addition, six studies enrolled community-based populations [8, 9, 12, 14, 21, 22], and the rest were controlled through hospital populations. Only two articles mentioned gene sequencing using a blind method [16, 20] (Table 1).

### 3.2. Study Characteristics

The characteristics of the studies are summarized in Table 1. Three studies were from Japan [4, 5, 19], one study was from Spain [16], seven studies were from China [6, 7, 20, 25–28], three studies were from England [13, 15], two studies were from the United States [21, 23], one study was from Israel [22], two studies were from Italy [9, 12], one study was from Croatia [10], one study was from Tunisia [8], one study was from the Czech Republic [18], one study was from Holland [24], two studies were from India [11, 14], and one study was from Iran [17]. The disease diagnosis standards of twelve studies were consistent with World Health Organization criteria [4, 6–9, 13, 14, 16, 23, 24, 27]; the disease diagnosis standards of seven studies had no description [11, 15, 19, 21, 22, 26, 28]; two studies were consistent with World Health Organization and American Heart Association criteria [5, 12]; one was consistent with American College of Cardiology and American Heart Association criteria [20], two studies were confirmed by coronarography [10, 25], and two were consistent with American College of Cardiology and European Society of Cardiology criteria [17, 18]. Eight studies on disease definition concerned coronary heart disease (CHD) or coronary artery disease (CAD) [8, 10, 11, 19, 25–27], fifteen were on myocardial infarction (MI) [4, 7, 9, 12–15, 17, 18, 21–24], and six concerned acute coronary disease (ACS) [5, 6, 16, 20]. The HPA-2a/b allele model and the dominant model each had fifteen studies that were selected. Eight studies were included in the GP VI T13254C allele model, and six studies were in the GP VI T13254C dominant model. Only five studies were included in the GP Ib*α* VNTR model because some research studies did not conform to the Hardy-Weinberg equilibrium. In the HPA-2a/b allele model, there were 2650 patients with CAD included, and 2762 patients with CAD in the HPA-2a/b dominant model. In the GP VI T13254C allele model, there were 2352 patients with CAD included, and 1595 patients with CAD were in the GP VI T13254C dominant model. In the GP Ib*α* VNTR gene model, there were 861 patients with CAD included.

### 3.3. Meta-Analysis Results

**Figure 2:**
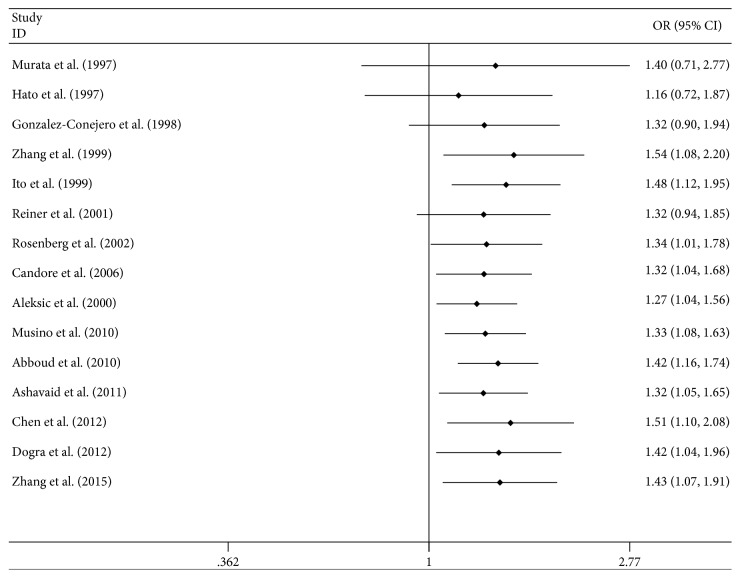
Cumulative meta-analysis of studies examining association between HPA-2a/b polymorphisms and risk of coronary artery disease.

Fifteen studies were included in the HPA-2a/b allele model and the dominant model. The association between HPA-2 polymorphism and CAD showed a significant difference utilizing the random-effects model in allele model (OR = 1.43, 95%CI = 1.07–1.91, *P* = 0.01) and dominant model (OR = 1.57, 95%CI = 1.08–2.28, *P* = 0.02) (Table 2). Cumulative meta-analysis showed that the difference was statistically significant since the study of Rosenberg et al. and significant difference tends to stability after this (Figure 2). However, subgroup analysis showed that this difference was not statistically significant among Indians.

Eight studies were included in the GP VI T13254C allele model and six studies in the GP VI T13254C dominant model. No significant association was demonstrated between the GP VI T13254C polymorphism and CAD in either the allele model (OR = 1.06, 95% CI = 0.82–1.37, random-effects model) or the dominant model (OR = 1.11, 95% = 0.93–1.31, fixed-effects model) (Table 3). In this meta-analysis, the studies by Gonzalez-Conejero et al. lacked the A allele, and Ni et al. lacked the D allele in both the CAD group and the control group. Therefore, only five studies were included in the GP Ib*α* VNTR polymorphism. The overall results from the different genetic analysis models identified no significant association between GP Ib*α* VNTR polymorphism and CAD (Table 3).

### 3.4. Heterogeneity Test

**Figure 3:**
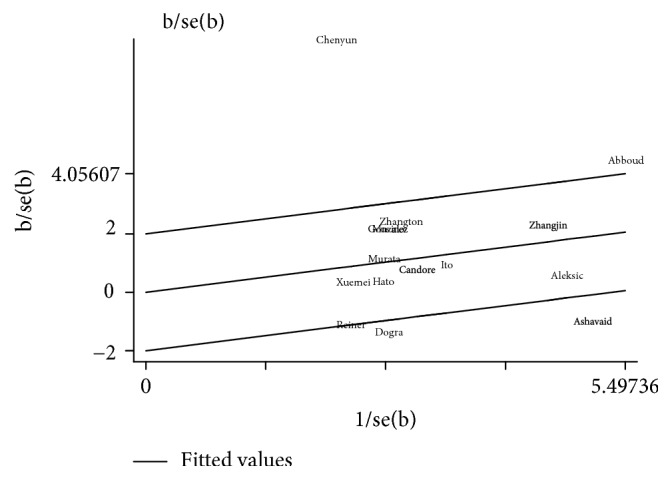
Galbraith plot for heterogeneity test of studies examining association between HPA-2a/b polymorphisms and risk of coronary artery disease.

A significant heterogeneity was present in the HPA-2a/b models (allele model *I*^2^ = 78%, dominant model *I*^2^ = 84%). Heterogeneity analysis revealed that the study of Chen and Zen from East Asians ethnic studies was the largest source of heterogeneity (Figure 3). In order to find out the source of heterogeneity in clinical and methodological presentation, a metaregression was performed to explore the source of heterogeneity in both the allele model and the dominant model, respectively. However, no clinical and methodological heterogeneity was found (*P* ≥ 0.05). This shows that the heterogeneity is derived from the statistical methods. We also found significant heterogeneity in the meta-analysis of the association between the GP VI T13254C allele model, the GP Ib*α* VNTR polymorphism, and CAD. Because less than 10 studies were included in the meta-analysis, a metaregression analysis was not performed to explore the source of heterogeneity.

### 3.5. Sensitivity Analysis

In the sensitivity analysis of the HPA-2 polymorphism and CAD in the allele models and dominant models, the ORs and 95% CIs were still 1.28 (1.04–1.58) and 1.30 (1.03–1.65), respectively, even after exclusion of the largest sources of heterogeneity from Chen and Zen. This fully shows that the meta-analysis results for the association between HPA-2a/b polymorphism and CAD have considerable stability. Meanwhile, this indicates that the result of Chen and Zen is the main factor leading to the increase in heterogeneity. Similarly, a sensitivity analysis did not change the results of the meta-analysis in the allele model or the dominant model for GP VI T13254C and the different genetic analysis models for GP Ib*α* VNTR.

### 3.6. Publication Bias

**Figure 4:**
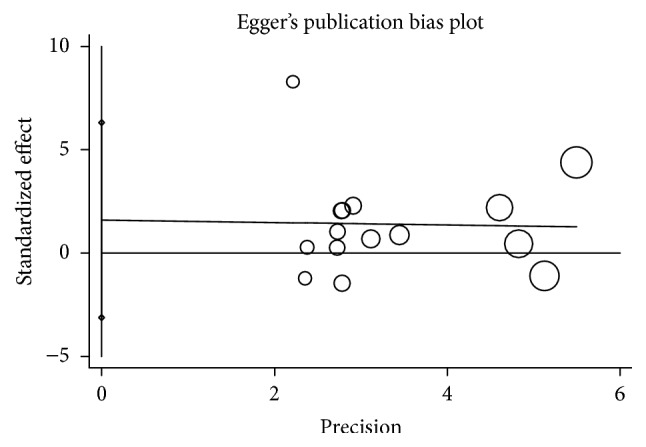
Publication bias of studies examining association between HPA-2a/b polymorphisms and risk of coronary artery disease.

Publication bias among the eligible studies was assessed by the Egger's test, and there was no publication bias in our meta-analysis (HPA-2a/b allele model: *P* = 0.767; HPA-2a/b dominant model: *P* = 1.000; GP VI T13254C allele model: *P* = 1.000; GP VI T13254C dominant model: *P* = 0.707; GP Ib*α* VNTR: omitted) (Figure 4).

## 4. Discussion

A total of twenty-five studies were included in our systematic review. The results indicated that the HPA-2a/b polymorphism was associated with CAD in both the allele model and dominant model. There was no evidence to suggest that polymorphisms of GP VI T13254C and GP Ib*α* VNTR were associated with CAD.

At the beginning of this century, a number of researchers conducted systematic reviews on the association of platelet membrane glycoprotein polymorphisms and risk of CAD. One of the most studied was the Pl^A1^/Pl^A2^ (HPA-1a/b) polymorphism of the platelet membrane glycoprotein receptor IIIa. Wu et al., Di Castelnuovo et al., and Burr et al. demonstrated meta-analysis results supporting a GP III a PlA1/PlA2 polymorphism associated with cardiovascular disease or coronary heart disease, although this correlation was rather weak. But in the years that followed, Ye et al. and Bentley et al. showed a meta-analysis whereby a platelet membrane glycoprotein polymorphism of PlA1/PlA2 (including GP Ib*α* Kozak-5T/C and GP Ia 807C/T) was associated with coronary heart disease, which was not statistically significant. This shows that the criteria for literature selection and meta-analysis method have a great influence on the results.

The main differences in the research methodology between this study and the meta-analyses reported above are as follows: (1) this study is limited to case control studies, and any retrospective or cohort studies were not included. (2) Studies were excluded that did not meet the Hardy-Weinberg equilibrium. (3) We used the Newcastle-Ottawa Scale (NOS) to evaluate the quality of the papers; this scale is mainly applied to a quality evaluation of the literature in case control and cohort studies. (4) A variety of genetic analysis models were used in combined-effects models (allele model, dominant model, and GP Ib*α* VNTR polymorphism using more complex gene analysis models).

This study still has some shortcomings. We failed to collect the unpublished literature, which is a type of study likely to avoid a publication bias. Due to the statistical analysis of a small part of the original documents using only the dominant model, no specific or calculated allele data, and the fact that we contacted relevant authors through e-mail and oftentimes did not receive a reply, a combined analysis of the allele model could not be carried out. The very strict inclusion and exclusion criteria resulted in a final statistical analysis of a smaller portion of the research, especially for GPIb*α*VNTR, which only included 5 papers. These weaknesses can only be compensated for by carrying out more comprehensive literature collection and more rigorous clinical trials.

## 5. Conclusion

We confirmed that the HPA-2a/b polymorphism is significantly associated with CAD and the HPA-2b allele and HPA-2ab+bb genotype are the genetic risk factors for CAD. We found no evidence to support the theory that the presence of GP VI T13254C and GP Ib*α* VNTR polymorphisms was related to an increased risk of CAD. However, in the future, we will need a larger sample size, a more appropriate population, and better genetic testing methods to verify this view.

## Figures and Tables

**Table 1:** Primary characteristics of included studies.

References	Location	Race	Diagnostic criteria	Gene	Age (yrs)		Disease definition	Male (%)	Source of controls	Sample	*P* for HWE
					Ca	Co		Ca/Co		Ca/Co	
Hato et al. 1997	Japan	2	WHO	1	54.0 ± 5.6	52.3 ± 7.0	MI	86/90	Hospital	88/100	0.340
Murata et al. 1997	Japan	2	No description	1	60.7 ± 8.7	49.1 ± 5.5	CAD	70/91	Hospital	91/105	0.367
Gonzalez-Conejero et al. 1998	Spain	1	WHO	1,3	62.9 ± 11.1	63.7 ± 10.9	ACS	73/73	Hospital	101/101	0.893
Ito et al. 1999	Japan	2	WHO, AHA	1,3	59.3 ± 10.0	51.0 ± 11.2	ACS	85/72	Hospital	158/169	0.912
Zhang et al. 1999	China	2	No description	1	59.2 ± 11.6	56.3 ± 10.7	MI	71/63	Hospital	100/104	0.364
Croft et al. 2001	England	1	WHO	2	61.9 ± 9.2	61.1 ± 9.1	MI	65/63	Hospital	289/292	0.181
Croft et al. 2001	England	1	WHO	2	61.5 ± 9.3	54.4 ± 11.8	MI	73/59	Hospital	236/182	0.972
Reiner et al. 2001	USA	1	No description	1	18–44	18–44	MI	NA	Community	68/346	0.763
Douglas et al. 2002	England	1	No description	3	59.1 ± 9.9	57.0 ± 10.6	MI	90/60	Hospital	88/168	0.952
Rosenberg et al. 2002	Israel	1	No description	1	42.5 ± 4.2	40.6 ± 4.1	MI	100/100	Hospital	100/119	NA
NI et al. 2004	China	2	ACC/AHA	3	60.6 ± 9.0	57.4 ± 11.0	ACS	62/62	Hospital	69/69	0.164
Kou et al. 2004	China	2	Coronarography	2	59.95 ± 6.31	58.76 ± 6.32	CAD	60/57	Hospital	179/164	0.718
Candore et al. 2006	Italy	1	WHO, AHA	1	<46	*∗*	MI	91/91	Community	105/110	0.671
Aleksic et al. 2000	Croatia	1	Coronarography	1	60.4 ± 9.3	57.5 ± 10.6	CAD	77/53	Hospital	402/202	0.284
Xue et al. 2008	China	2	WHO	1	NA	NA	CAD	NA	Hospital	212/106	NA
Abboud et al. 2010	Tunisia	1	WHO	1	55.5 ± 10.6	54.1 ± 9.4	CAD	72/76	Community	247/316	0.996
Motovska et al. 2010	Czech	1	ACC/ESC	2	47.78 ± 6.13	63.66 ± 9.47	MI	78/34	Hospital	105/132	NA
Musino et al. 2010	Italy	1	WHO	1	47.2	50	MI	86/67	Community	71/150	0.816
Snoep et al. 2010	Holland	1	WHO	2	57 (49–64)	59 (50–66)	MI	100/100	Hospital	545/643	0.480
Shaffer et al. 2011	USA	1	WHO	2	NA	NA	MI	77/77	Community	652/625	NA
Ashavaid et al. 2011	India	3	No description	1,3	58.6 ± 10.4	43.0 ± 11.3	CAD	80/54	Hospital	446/473	0.999
Dogra et al. 2012	India	3	WHO	1	36.4 ± 4.5	31.1 ± 6.0	MI	96/76	Community	184/350	0.340
Chen and Zen 2012	China	2	WHO	1	61.05 ± 10.27	60.74 ± 6.80	MI	79/79	Hospital	86/85	0.543
Kazemi et al. 2012	Iran	1	ACC/ESC	2	46.3 ± 5.2	44.7 ± 6.8	MI	84/41	Hospital	100/100	0.061
Sun et al. 2012	China	2	No description	2	75.96 ± 7.45	71.72 ± 8.31	CAD	68/63	Hospital	246/185	0.852
Zhang et al. 2015	China	2	WHO	1	62.5 ± 11.9	60.1 ± 10.2	ACS	71/59	Hospital	403/500	0.973

Race: (1) Caucasian; (2) East Asians; (3) Indian. Gene: (1) HPA-2; (2) GP VI T13254C; (3) GP Ib*α* VNTR. Ca, case group; Co, control group. CAD, coronary artery disease; MI, myocardial infarction (acute or chronic); ACS, acute coronary syndrome. ^*∗*^Matching with the control; WHO, World Health Organization; AHA, American Heart Association; ESC, European Society of Cardiology.

**Table 2:** Meta-analysis of studies examining association between HPA-2a/b polymorphisms and risk of coronary artery disease according to race.

Genotype	Number of trails	Heterogeneity		Effect model	Meta-analyses		
		*I* ^2^	*P* value		ORs	95% CIs	*P* value
Allelic model							
All	15	78	<0.01	Random	1.43	1.07–1.91	0.01
Race							
Caucasian	7	48	0.07	Fixed	1.40	1.06–1.84	0.02
East Asians	6	84	<0.01	Random	1.95	1.09–3.50	0.03
Indian	2	0%	0.48	Fixed	0.75	0.55–1.04	0.08
Dominant model							
All	15	84	<0.01	Random	1.57	1.08–2.28	0.02
Race							
Caucasian	6	63	0.02	Random	1.46	1.00–2.14	0.05
East Asians	7	89	<0.01	Random	2.23	1.06–4.71	0.04
Indian	2	0%	0.45	Fixed	0.75	0.54–1.05	0.10

**Table 3:** Meta-analysis of studies examining association between GP VI T13254C, GP Ib*α* VNTR polymorphisms and risk of coronary artery disease.

Genotype	Number of trails	Heterogeneity		Effect model	Meta-analyses		
		*I* ^2^	*P* value		OR	95% CIs	*P* value
GP VI T13254C allele model	8	73	<0.01	Random	1.0	0.82–1.37	0.66
GP VI T13254C dominant model	6	4	0.39	Fixed	1.1	0.93–1.31	0.24
GP Ib*α* VNTR A allele model	5	0	0.47	Fixed	1.1	0.70–1.91	0.58
GP Ib*α* VNTR B allele model	5	51	0.08	Random	1.0	0.72–1.59	0.74
GP Ib*α* VNTR C allele model	5	57	0.06	Random	1.0	0.80–1.32	0.86
GP Ib*α* VNTR D allele model	5	0	0.43	Fixed	1.1	0.93–1.32	0.25
GP Ib*α* VNTR A allele dominant model	5	0	0.49	Fixed	1.0	0.60–1.81	0.88
GP Ib*α* VNTR B allele dominant model	5	67	0.02	Random	1.2	0.70–2.20	0.46
GP Ib*α* VNTR C allele dominant model	5	34	0.19	Fixed	0.8	0.58–1.31	0.50
GP Ib*α* VNTR D allele dominant model	5	0	0.80	Fixed	1.0	0.82–1.25	0.91
GP Ib*α* VNTR BC codominant model	5	76	<0.01	Random	1.3	0.64–2.74	0.44
GP Ib*α* VNTR CC codominant model	5	58	0.05	Random	0.9	0.64–1.29	0.59
